# Targeting Arginase 1 but Not Arginase 2 Protects from Myocardial Ischemia–Reperfusion Injury via Nitric Oxide Signaling by Red Blood Cells in Type 2 Diabetes

**DOI:** 10.3390/antiox15010058

**Published:** 2026-01-01

**Authors:** Jiangning Yang, Yahor Tratsiakovich, Renhai Cao, Ali Mahdi, Gianluigi Pironti, Tong Jiao, Rawan Humoud, Eftychia Kontidou, John Tengbom, Aida Collado, Zhichao Zhou, Yihai Cao, Eleonore Köhler, Adam E. Mullick, John Pernow

**Affiliations:** 1 Department of Medicine, Unit of Cardiology, Karolinska Institutet, 17177 Stockholm, Swedenjohn.pernow@ki.se (J.P.); 2 Center for Molecular Medicine, Karolinska University Hospital, 17176 Stockholm, Sweden; 3 Department of Cardiology, Karolinska University Hospital, 17176 Stockholm, Sweden; 4 Department of Physiology and Pharmacology, Karolinska Institutet, 17177 Stockholm, Sweden; 5 Department of Microbiology, Tumor and Cell Biology, Karolinska Institutet, 17177 Stockholm, Sweden; 6 Department of Anatomy and Embryology, Maastricht University, 6200 MD Maastricht, The Netherlands; 7 Ionis Pharmaceuticals Inc., Carlsbad, CA 92010, USA

**Keywords:** arginase, nitric oxide, ischemic tolerance, type 2 diabetes, sex

## Abstract

Background: Arginase influences cardiac tolerance to ischemia–reperfusion by modulating nitric oxide (NO) signaling. In type 2 diabetes (T2D), elevated arginase activity may worsen ischemic injury through red blood cells (RBCs), but the specific roles of arginase isoforms are unclear. Methods: C57BL/6 and db/db mice were pretreated with *ARG1* or *ARG2* antisense oligonucleotides (ASO) for six weeks. Conditional *ARG1* knockout (*ARG1^fl/fl^*/*Tie2Cre^tg/−^*) and wild-type littermates were also studied. Mice underwent coronary artery ligation and reperfusion in vivo for infarct size assessment. In ex vivo experiments, buffer-perfused hearts were subjected to global ischemia–reperfusion with or without RBCs to evaluate recovery of left ventricular developed pressure (LVDP). Results: *ARG1* knockdown, but not *ARG2*, improved post-ischemic recovery of LVDP in isolated hearts. RBCs from *ARG1* ASO-treated mice enhanced recovery in wild-type hearts, while *ARG1* knockout reduced infarct size compared with controls. Cardioprotection was abolished by NO synthase inhibition. RBCs from male and female *ARG1* knockout mice improved LVDP recovery compared with RBCs from wild-type mice. In T2D mice, impaired recovery was restored by *ARG1* ASO or RBCs from *ARG1* ASO-treated T2D mice. Conclusions: Arginase 1, but not arginase 2, limits cardiac tolerance to ischemia–reperfusion and contributes to increased vulnerability in T2D.

## 1. Introduction

It is well established that nitric oxide (NO) plays a critical role in cardiovascular regulation and protection [1,2,3,4,5]. Reduced bioavailability of NO has been implicated in the pathogenesis of cardiovascular disease including coronary artery disease and myocardial infarction [6]. One key mechanisms leading to reduced NO bioavailability is increased activity of the enzyme arginase, which competes with endothelial nitric oxide synthase (eNOS) for their common substrate L-arginine [7,8,9,10,11]. Elevated arginase activity reduces NO-dependent signaling, promotes endothelial dysfunction, and ultimately worsens myocardial injury following ischemia–reperfusion [12,13].

Arginase exists in the two isoforms arginase 1 and 2 which are encoded by the separate genes *ARG1* and *ARG2* [8,14,15]. Arginase 1 is primarily localized in the cytoplasm, whereas arginase 2 is predominantly mitochondrial. Both isoforms are presented in the cardiovascular system. It is of interest that arginase activity and expression are increased in the cardiovascular system in type 2 diabetes (T2D) [12,16,17], leading to attenuated NO bioavailability [18] and blunted NO-mediated cardioprotective effects [19,20,21]. Notably, pharmacological inhibition of arginase has been shown to restore both cardiac and endothelial function in T2D [12,22]. It is therefore tempting to speculate that arginase contributes to poor cardiovascular outcomes in T2D [21].

Our previous study [12,23] suggests that arginase in red blood cells (RBCs) plays a particularly important role in the regulation of NO bioavailability and oxidative stress, thereby contributing to myocardial ischemia–reperfusion injury. Inhibition of RBC arginase was shown to improve cardiac ischemic tolerance as determined by post-ischemic recovery of left ventricular developed pressure (LVDP). This effect may be of particular importance in T2D, a condition associated with increased RBC arginase activity and oxidative stress [12]. Although RBC-derived arginase 1 is considered a key contributor in limiting cardiac ischemic tolerance, the specific role of the two arginase isoforms in other cell types, e.g., endothelial cells (ECs), remains unclear.

A major limitation in previous studies investigating the pathophysiological role of arginase has been the lack of isoform-specific inhibitors. It has therefore not been possible to determine the relative contribution of the individual arginase isoforms for cardiovascular disease in T2D. Transcriptional modification methods to silence and knockdown *ARG1* or *ARG2* or genetic deletions have emerged as efficient tools to clarify their roles. However, a global knockout (KO) of *ARG1* resulted in a lethal phenotype [24]. Therefore, conditional deletion of the *ARG1* gene is a reasonable choice for studying the specific role of arginase 1 in the development of cardiovascular disease [25].

The present study was designed to determine the specific role of the two arginase isoforms using knockdown and genetic deletion of arginase, with special focus on the role of RBC arginase and the therapeutic effect of targeting arginase in myocardial ischemia–reperfusion in T2D.

## 2. Materials and Methods

### 2.1. Ethic Approval and Consent to Participate

The study was approved by the regional Ethics Committee for laboratory animal experiments in Stockholm and conforms to the Guide for the Care and Use of Laboratory Animals published by the US National Institutes of Health (NIH Publication No. 85-23, revised 1996).

### 2.2. Animals

Male C57BL/6J and *db*/*db* mice were purchased from Janvier Labs (Le-Genest Saint-Isle, France). Conditional arginase 1 knockout *ARG1^fl/fl^*/*Tie2Cre^tg/−^* (*ARG1* KO) mice and *ARG1^fl/fl^*/*Tie2Cre^−/−^* littermates (*ARG1* WT), previously described in detail [26], were used for the experiments at the age of 3–7 months. This conditional KO model lacks arginase 1 in EC and hematopoietic cells. All animals were kept at the animal facility in Karolinska Institutet, Stockholm, Sweden, with a 12/12 h dark/light cycle, and were given food and water *ad libitum*.

### 2.3. Genotyping

Genotyping of *ARG1* KO mice was performed by using the KAPA HotStart Mouse Genotyping Kit (KAPA Biosystems, Cape Town, South Africa).

### 2.4. Administration of Arginase1/2 Antisense Oligonucleotides

Arginase 1 and arginase 2 antisense oligonucleotides (*ARG1* ASO, sequence ATACTACTTCTTAAGG and *ARG2* ASO, sequence TTAATTAATGCACCAG) were kindly provided by Ionis Pharmaceuticals (Carlsbad, CA, USA). These ASOs incorporate constrained-ethyl (cEt) sugar modifications to improve target RNA binding affinity, potency, stability and tissue distribution relative to earlier generations (e.g., MOE chemistry) [27].

C57BL/6J and T2D *db*/*db* mice were treated with the *ARG1* or *2* ASO by i.p. injections (50 mg/kg) once weekly for six weeks. The dose was based on preliminary optimization experiments showing robust reduction in arginase 1 and 2 with no adverse effects on liver or kidney function, sites of high ASO distribution particularly susceptible to nonselective and poorly tolerated ASOs [28]. Considering that the lifespan of mouse RBCs is approximately 40 days, a six-week treatment period was selected to ensure effective knockdown of arginase expression in circulating RBCs (Supplemental Figure S1).

### 2.5. Heart Isolation and Perfusion

Isolated mouse hearts were perfused in a Langendorff system (AstraZeneca, Mölndal, Sweden) as described previously [23,29,30]. Briefly, the mice were anesthetized with pentobarbital (50 mg/kg i.p.). Following injection of heparin (250 IU i.p.), the hearts were excised and the ascending aorta was cannulated and perfused with oxygenated (5% CO_2_, 95% O_2_) modified Krebs–Henseleit (KH) buffer (in mM: NaCl 118.5, NaHCO_3_ 25.0, KCl 4.7, KH_2_PO_4_ 1.2, MgSO_4_ 1.2, glucose 11.1, and CaCl_2_ 2.4) at 70 mmHg and 37 °C. A balloon-tipped catheter connected to a pressure transducer was inserted into the left ventricle for the recording of LVDP. The balloon was inflated to give a baseline left ventricular end-diastolic pressure of 4–10 mmHg.

### 2.6. Isolation of RBCs

Whole blood was collected from the thoracic cavity after the excision of the heart. RBCs were isolated by centrifugation at 1000× *g* and 4 °C for 10 min followed by removal of plasma and the buffy coat. The RBCs were then washed with KH buffer and centrifuged 3 times as previously described [12,23,31]. This results in >99% removal of leukocytes and platelets [23]. In additional experiments, RBCs were passed through a leukocyte filter (Acrodisc WBC 25 mm PSF, Pall Corporation, Port Washington, NY, USA) to avoid contamination from any remaining cells.

### 2.7. Arginase Activity

Arginase activity was determined using a colorimetric assay as previously described [23]. Briefly, mouse RBCs, kidneys, or livers were lysed using radioimmunoprecipitation (RIPA) lysis buffer (Amresco, Solon, OH, USA) containing a protease inhibitor cocktail (Roche, Mannheim, Germany) followed by centrifugation for 20 min at 10,000× *g* at 4 °C. The supernatant was collected, and the total protein content of the extracts was quantified using a bicinchoninic acid protein assay kit (Pierce™ BCA Protein Assay Kit, Thermo Fisher Scientific, Rockford, IL, USA). Lysate (50 μL) was added to 75 μL of 10 mM MnCl_2_ (mixed with TRIS 50 mM, pH 7.5). Arginase was activated by heating the mixture at 56 °C for 10 min. Hydrolysis of arginine was achieved by adding 50 μL of L-arginine (0.5 M dissolved in 50 mM TRIS, pH 9.7), and then incubated at 37 °C for 30 min. The reaction was stopped by adding 400 μL of acid mixture (H_2_SO_4_:H_3_PO_4_:H_2_O = 1:3:7). After adding 25 μL of α-isonitrosopropiophenone (9% in ethanol), the mixture was incubated at 100 °C for 60 min. The mixture was loaded onto a centrifugal filter and centrifuged for 5 min at 5000× *g* at room temperature. The concentration of urea in the filtrate was determined in a microplate reader (Wallac 1420 VICTOR2™, PerkinElmer Life Sciences, Boston, MA, USA) at 540 nm. Arginase activity was calculated as urea production (mmol/mg protein/min).

### 2.8. Quantitative PCR

Quantitative PCR was performed as previously described. Briefly, total RNA was extracted from murine bone marrow cells using RNeasy^®^ Mini Kit (QIAGEN GmbH, Hilden, Germany). Isolated RNA was subsequently reverse-transcribed using a High-Capacity RNA-to-cDNA kit (Applied Biosystems (Waltham, MA, USA), Thermo Fisher Scientific). PCR amplification was performed in a 7900 HT real-time PCR system (Applied Biosystems, Thermo Fisher Scientific) using TaqMan^®^ Universal PCR Master Mix (Applied Biosystems, Thermo Fisher Scientific) and TaqMan^®^ Gene Expression Assays (Arg I Rn00691090_m1, GAPDH Rn01775763). All samples were measured in duplicates. Results were normalized to the equal mass of total RNA as well as the Ct values of GAPDH. The relative amount of mRNA was calculated by the 2^−ΔΔCt^ method and presented as fold change.

### 2.9. Immunohistochemistry

Whole mount immunohistochemical staining of tissue samples was performed according to our previously published methods [32]. Briefly, liver and kidney tissues were fixed with 4% (*w*/*v*) paraformaldehyde overnight and cut into small pieces. Tissues were digested with 20 mM proteinase K in 10 mM Tris buffer (pH 7.5) for 5 min, followed by incubation with 100% (*v*/*v*) methanol for 30 min. Tissues were washed with phosphate-buffered saline (PBS) and incubated overnight at 4 °C in PBS containing 5% monkey serum and 0.3% Triton X-100, followed by incubation overnight with various combinations of a goat anti-mouse CD31 antibody (AF3628, R&D Systems, Minneapolis, MN, USA), a rabbit polyclonal anti-arginase 1 antibody (1:100, #HPA003595, Atlas, Sigma-Aldrich, St. Louis, MO, USA), and a rabbit polyclonal anti-arginase 2 antibody (1:100, #HPA000663, Atlas, Sigma-Aldrich). After rinsing, the tissues were incubated overnight with an Alexa Fluor 647-labeled donkey anti-mouse or anti-goat antibody and an Alexa Fluor 555-labeled donkey anti-rabbit antibody (Invitrogen, Waltham, MA, USA). After washing, stained tissue samples were mounted using a Vectashield mounting medium (Vector Laboratories, Newark, CA, USA), and images were captured by confocal microscopy (Nikon C1 confocal microscope; Nikon Corporation, Shinagawa, Tokyo, Japan).

### 2.10. Western Blot

Samples were lysed using RIPA lysis buffer (VWR International, Radnor, PA, USA), sonicated 5 times at room temperature for 5 min each, and centrifuged at 1000× *g* for 1 min. The protocol of Western blot was previously described in detail [23]. Briefly, the total protein content of the extracts was quantified by a bicinchoninic acid protein assay kit (Pierce Biotechnology, Life Technologies, Rockford, MA, USA). The proteins were separated on 10% SDS gel and transferred onto 0.45 μm nitrocellulose blotting membranes (Amersham, Buckinghamshire, UK). Membranes were blocked with 5% milk for 1 h at room temperature and incubated overnight at 4 °C with a rabbit polyclonal anti-human arginase-1 primary antibody (1:1000, #HPA003595, Atlas, Sigma-Aldrich, St. Louis, MO, USA), or a rabbit polyclonal anti-arginase 2 antibody (1:1000, #HPA000663, Atlas, Sigma-Aldrich, St. Louis, MO, USA), or GAPDH (1:2500, # SAB5600208 Sigma-Aldrich, St. Louis, MO, USA) followed by incubation with a goat anti-rabbit secondary antibody for 1 h at room temperature (1:20,000 dilution, catalog No. 926-32211, LI-COR IRDye^®^ 800CW; LI-COR Biosciences, Lincoln, NE, USA).

### 2.11. Isolated Heart Studies

After the start of perfusion [23], all hearts were allowed to stabilize for at least 30 min, and baseline LVDP was registered. Global ischemia was induced by clamping the aortic inflow. The duration of global ischemia was 40 min, and reperfusion was 60 min. At the start of ischemia, the RBC suspension (Hct ~40%, 0.4 mL) was injected into the coronary circulation via a side arm of the perfusion system. The RBCs were administered with vehicle, the NOS inhibitor N-Nitro-L-arginine methylester (L-NAME, 100 μM, Sigma-Aldrich), or the inhibitor of soluble guanylate cyclase (sGC) 1H- [1,2,4] oxadiazolo [4,3,-a] quinoxalin-1-one (ODQ; 5 μM, Sigma-Aldrich). The concentrations were based on previous studies [12,31]. Any preparation that showed signs of hemolysis was excluded.

### 2.12. In Vivo Ischemia–Reperfusion Studies

*ARG1* WT and *ARG1* KO mice were used for in vivo myocardial ischemia–reperfusion experiments. Mice were anesthetized with pentobarbital 100 mg/kg i.p. followed by additional i.v. infusion when needed to maintain anesthesia during the experiment. Rectal temperature was maintained at 37.5–38.5 °C by a heated operation table. Mice were tracheotomized, intubated, and ventilated with a mixture of air (0.7 L/min) and oxygen (0.3 L/min) by a murine ventilator (Ugo Basile SRL, Gemonio, Italy) at a rate of 105 strokes/min, 0.4 mL/kg tidal volume. Heart rate and ST-segment changes were monitored by continuous recording of electrocardiogram (ECG) on a personal computer equipped with PharmLab V5.0 (AstraZeneca R&D, Mölndal, Sweden). The second standard ECG lead was used by applying needle electrodes to respective extremities. A Zeiss Op-Mi6 microscope (Oberkochen, Germany) was used to perform fine surgical procedures. The left jugular vein was cannulated with a PE-10 catheter for i.v. administrations. The heart was exposed via a left thoracotomy, and an Ethicon 6-0-gauge silk ligature was placed around the left coronary artery. After completion of the surgical preparation, the mice were allowed to stabilize for at least 15 min before being randomized into different experimental groups (see below). Ischemia was induced by tightening the ligature around the left coronary artery. A 4 mm long piece of PE-10 tubing was placed between the artery and silk to protect the vessel from mechanical damage. Successful occlusion was associated with immediate ST-segment elevation on the ECG, increased heart rate, and cyanosis of the myocardial area at risk. Reperfusion was initiated after 30 min of ischemia by removal of the ligature and was maintained for 2 h. The reperfusion was associated with the disappearance of the cyanotic color of the myocardium and normalization of the ST segment on the ECG. To avoid a decrease in the temperature in the thoracic cavity, it was thoroughly covered during the experiment.

*ARG1* KO and WT mice were randomized to the following treatments given i.v. 15 min prior to coronary artery occlusion: saline (WT, *n* = 6; *ARG1* KO, *n* = 6); the arginase inhibitor N^ꞷ^-hydroxy-nor-L-arginine (nor-NOHA, 100 mg/kg, Bachem, Bubendorf, Switzerland; WT; *n* = 5) or the NOS inhibitor N^G^-monomethyl-L-arginine monoacetate (L-NMMA, 10 mg/kg, Alexis Biochemicals Lausen, Switzerland; *ARG1* KO; *n* = 5). The doses were based on previous studies [33]. L-NMMA has previously been shown not to affect infarct size *per se* [23] and in study.

### 2.13. Determination of Infarct Size and Collection of Myocardial Samples

Infarct size was measured as previously described [33]. Briefly, after 2 h of reperfusion, the coronary artery was reoccluded, and 0.6 mL of 2% Evans blue dye (Sigma-Aldrich) solution was injected into the left jugular vein to stain non-ischemic myocardium for the determination of the area at risk. The mice were euthanized by exsanguination, and the hearts were extracted. Each heart was shortly frozen at −20 °C to facility cutting and cut into 7–9 slices perpendicular to the base-apex axis. All slices were weighed, scanned from both sides for the determination of the area at risk, and put in 1% triphenyltetrazolium chloride (TTC, Sigma-Aldrich) solution for 15 min at 37 °C and put in 4% formaldehyde to distinguish viable from necrotic myocardium. After 24 h of incubation in 4% formaldehyde, slices were scanned again from both sides. Following Evans Blue and TTC staining, heart slices were coded. The quantification of the area at risk and infarct area was performed by an investigator blinded to the group assignments using planimetry of computer images (Adobe Photoshop CS 2; Adobe Systems, CA, USA).

### 2.14. Transthoracic Echocardiography

Cardiac function was non-invasively monitored by transthoracic echocardiography using a high-resolution imaging system (Philips HDI 5000, Philips Medical Systems, Bothell, WA, USA) with a CL-15 7 scanning head (Philips Medical Systems, Bothell, WA, USA). Briefly, WT mice treated with vehicle (control) or ASO (*n* = 6 WT Con and *n* = 6 WT ASO) and *db*/*db* mice treated with vehicle or *ARG1* ASO (*n* = 4 *db*/*db* Con and *n* = 4 *db*/*db* ASO) were anesthetized with isoflurane (2%) and laid on a heating pad (37 °C). The hair from the chest was removed using a depilatory cream. After echocardiography images acquisition, the mice were maintained on a heating pad (37 °C) without anesthesia until their recovery. M-mode imaging of the left ventricle short axis was analyzed, and cardiac contractility was expressed as % left ventricular fractional shortening using the following formula: LVd-LVs/LVd × 100, where LVd and LVs stand for left ventricle diastole and systole, respectively. Heart rate, posterior wall, and interventricular septum thickness were also measured.

### 2.15. Statistical Analysis

Data is presented as mean ± SD. Two-way ANOVA with Bonferroni post hoc test was used for multiple comparisons of heart rate in vivo and LVDP ex vivo. One-way ANOVA followed by the Bonferroni post hoc test was used for comparisons of area at risk and infarct size in vivo. Unpaired *t*-test was used for comparisons between two groups. A *p*-value < 0.05 was considered significant. All analyses were performed using GraphPad Prism™ 7.05 software (GraphPad Inc., San Diego, CA, USA).

## 3. Results

### 3.1. ARG1 ASO Reduces Arginase 1 Expression and Induces Cardiac Protection

**Figure 1:**
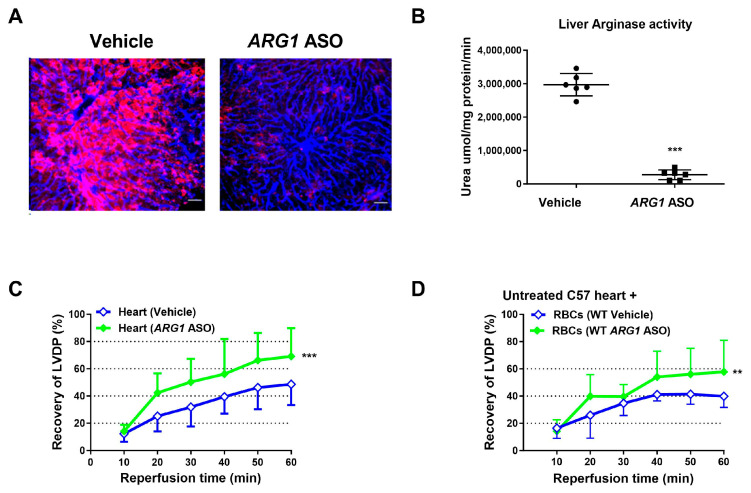
Arginase 1 anti-sense oligonucleotide reduces arginase 1 expression and induces cardiac protection. (**A**) Immunohistochemical images of liver from mice treated with vehicle or arginase 1 (*ARG1*) anti-sense oligonucleotide (ASO) for 6 weeks. Sections were stained with anti-arginase 1 (red) and anti-CD31 (blue). Scale bar = 50 μm. (**B**) Hepatic arginase activity in vehicle- and *ARG1* ASO-treated mice. (**C**,**D**) Recovery of left ventricular developed pressure (LVDP) in isolated hearts subjected to 40 min ischemia and 60 min reperfusion. (**C**) Buffer-perfused hearts from *ARG1* ASO-treated mice (*n* = 5) or vehicle (*n* = 10). (**D**) RBCs from *ARG1* ASO-treated mice (*n* = 9) or vehicle (*n* = 5) were administered at ischemia onset to isolated hearts. Data are shown as means and SD. ** *p* < 0.01 and *** *p* < 0.001 denote significant differences vs. vehicle, analyzed by unpaired *t*-test (**B**) or two-way ANOVA (**C**,**D**).

The roles of arginase 1 and arginase 2 were assessed using ASO administration in WT mice. Since the liver expresses predominantly arginase 1 and the kidney arginase 2 [34], these tissues were used for the determination of altered expression of arginase 1 and 2, respectively. Treatment with *ARG1* ASO resulted in a marked reduction in arginase 1 expression and enzymatic activity in the liver (Figure 1A,B), whereas renal arginase 2 expression remained unaffected (Supplemental Figure S2). Body weight was not altered by *ARG1* ASO treatment (Supplemental Figure S3). Echocardiographic evaluation revealed that *ARG1* ASO administration did not affect cardiac contractility or heart rate (Supplemental Figure S4A,B), nor did it influence systolic and diastolic cardiac dimensions (Supplemental Figure S4C,D) or left ventricular wall thickness (Supplemental Figure S4E,F) in vivo.

The effect of *ARG1* knockdown on tolerance to myocardial ischemia–reperfusion was investigated in isolated perfused hearts. Recovery of LVDP during reperfusion was significantly greater in hearts from mice given *ARG1* ASO than in hearts from vehicle-treated mice (Figure 1C). Of further importance, RBCs from *ARG1* ASO mice administered to the isolated hearts from untreated mice markedly improved cardiac post-ischemic LVDP in comparison with RBCs from vehicle-treated mice (Figure 1D). Collectively, these results suggest an important negative impact of arginase 1 on cardiac tolerance to ischemia–reperfusion.

### 3.2. ARG2 ASO Failed to Induce Cardiac Protection

**Figure 2:**
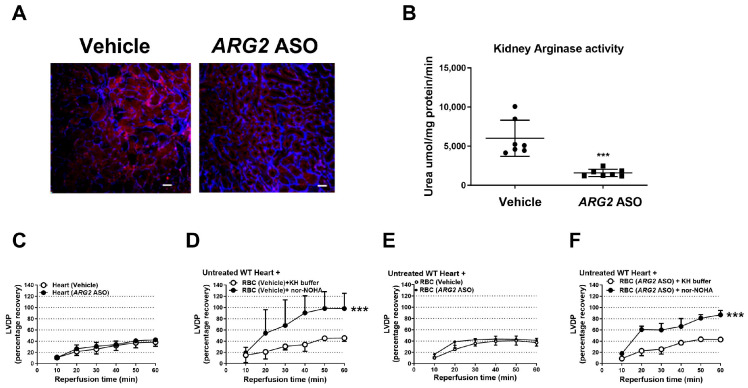
*ARG2* ASO fails to induce cardiac protection. (**A**) Immunohistochemical images of kidneys from mice treated with vehicle or *ARG2* ASO. Sections were stained with anti-arginase 2 (red) and anti-CD31 (blue). Scale bar = 50 μm. (**B**) Renal arginase activity in vehicle- and *ARG2* ASO-treated mice. (**C**–**F**) Recovery of LVDP in isolated hearts subjected to 40 min ischemia and 60 min reperfusion. (**C**) Buffer-perfused isolated hearts from *ARG2* ASO-treated mice (*n* = 6) or vehicle (*n* = 7). (**D**) RBCs from *ARG2* ASO-treated mice (*n* = 6) or vehicle (*n* = 7) administered to isolated WT hearts at ischemia onset. (**E**) RBCs from vehicle-treated mice incubated with the arginase inhibitor nor-NOHA (1 mM, *n* = 4) or vehicle (KH buffer, *n* = 4) before administration to WT hearts. (**F**) RBCs from *ARG2* ASO-treated mice incubated with nor-NOHA (1 mM, *n* = 4) or vehicle before administration. Data are shown as means and SD. *** *p* < 0.001 vs. vehicle (unpaired *t*-test or two-way ANOVA). Abbreviations as in Figure 1.

Administration of the *ARG2* ASO induced marked downregulation of arginase 2 (Figure 2A and Supplemental Figure S5A) but not arginase 1 expression in the kidney (Supplemental Figure S5B). Kidney arginase activity was also markedly decreased by *ARG2* ASO administration (Figure 2B). Body weight was not altered by the *ARG2* ASO treatment (Supplemental Figure S6). The recovery of LVDP following ischemia in isolated hearts from *ARG2* ASO-treated mice was not different from that in hearts from vehicle-treated mice (Figure 2C, Supplemental Figure S7A). Furthermore, administration of RBCs from mice treated with *ARG2* ASO did not affect recovery of LVDP (Figure 2D, Supplemental Figure S7B). By contrast, pharmacological inhibition of RBC arginase significantly improved post-ischemic recovery of LVDP (Figure 2E). Importantly, this was observed using RBCs derived from either control mice or *ARG2* ASO-treated mice (Figure 2F), supporting the notion that inhibition of arginase 1 in RBCs protects against cardiac ischemia–reperfusion injury.

### 3.3. In Vivo Conditional ARG1 KO Protects Against Myocardial Ischemia–Reperfusion Injury

According to the observations above, knockdown of *ARG1*, but not *ARG2*, induces protection against cardiac ischemia–reperfusion. To further explore the importance of arginase 1, a conditional KO mouse model, in which arginase 1 was specifically deleted in hematopoietic and endothelial cells, was used to identify the role of arginase 1. *ARG1* gene expression in bone marrow cells and arginase activity in RBCs were markedly lower in *ARG1* KO mice than in WT mice (Supplemental Figures S8 and S9). There were no significant differences in heart rate among mice included in the in vivo experiments (Supplemental Table S1).

**Figure 3:**
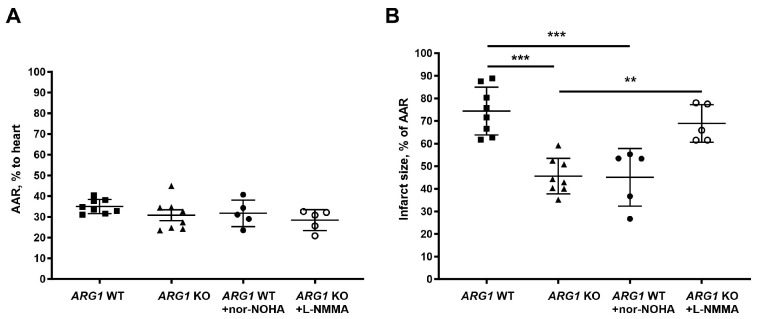
In vivo conditional *ARG1* knockout protects against myocardial ischemia–reperfusion injury. (**A**) Area at risk (AAR) and (**B**) infarct size after 30 min of ischemia and 2 h of reperfusion in wild-type mice given saline (WT, *n* = 6), conditional *ARG1* knockout mice given saline (*ARG1* KO), WT mice given the arginase inhibitor nor-NOHA (WT + nor-NOHA, *n* = 5), and *ARG1* KO mice given the NOS inhibitor L-NMMA (*ARG1* KO + L-NMMA, *n* = 5). Data are presented as mean ± SD. ** *p* < 0.01; *** *p* < 0.001 denote significant differences between groups by one-way ANOVA. Abbreviations as in Figure 1.

There were no significant differences in the area at risk between any of the groups (Figure 3A). Infarct size was significantly smaller in *ARG1* KO mice (45.6 ± 7.9%) than in WT mice (74.4 ± 10.1%) (Figure 3B). Administration of the arginase inhibitor nor-NOHA to WT mice significantly reduced infarct size to 45.1 ± 12.7% from 74.4 ± 10.6% in vehicle-treated mice (Figure 3B). The reduction in infarct size induced by administration of the arginase inhibitor in WT mice was comparable to that observed in *ARG1* KO mice. These data suggest that arginase 1 in endothelial cells or hematopoietic cells is of importance for cardiac injury during ischemia–reperfusion. The NOS inhibitor L-NMMA abolished the cardioprotective effect in *ARG1* KO mice (Figure 3B), suggesting that deficiency in arginase 1 leads to cardioprotection via a NOS-dependent mechanism.

### 3.4. RBC-Arginase 1 Is Important for Cardiac Protection

**Figure 4:**
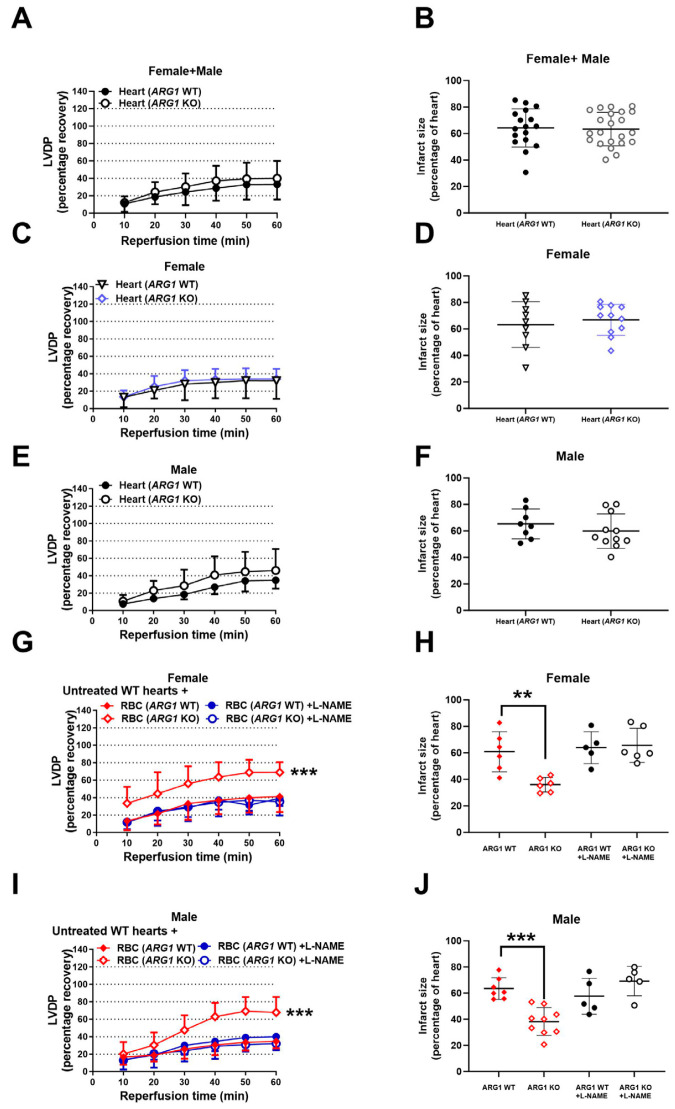
RBC-arginase 1 is essential for cardiac protection. Recovery of LVDP and infarct size in isolated hearts subjected to 40 min ischemia and 60 min reperfusion from*ARG1* conditional KO or WT mice. (**A**,**B**) Hearts from male and female *ARG1* KO and WT mice. (**C**,**D**) Hearts from female *ARG1* KO and WT mice. (**E**,**F**) Hearts from male *ARG1* KO and WT mice. (**G**,**H**) RBCs from female WT or *ARG1* KO mice, with or without the NOS inhibitor L-NAME (100 μM, *n* = 6), administered to isolated male WT hearts at ischemia onset. (**I**,**J**) RBCs from male WT or *ARG1* KO mice, with or without L-NAME (100 μM), administered to isolated male WT hearts at ischemia onset. Data are presented as means and SD. ** *p* < 0.01, *** *p* < 0.001 vs. vehicle by one- or two-way ANOVA. Abbreviations as in Figure 1 and Figure 2.

To identify the cellular origin of arginase 1 responsible for reduced tolerance to ischemia–reperfusion, the isolated perfused heart model was applied. Post-ischemic recovery of LVDP (Figure 4A) and infarct size (Figure 4B) in isolated hearts from *ARG1* KO mice did not differ from hearts from WT mice when perfused with KH buffer only, indicating that endothelial cell arginase 1 is not involved in the regulation of cardiac tolerance to ischemia–reperfusion. Furthermore, there was no difference between female and male mice regarding LVDP or infarct size in hearts from *ARG1* KO and WT mice (Figure 4C–F). By contrast, administration of RBCs from either female or male *ARG1* KO mice markedly improved post-ischemic functional recovery (Figure 4G,I) and reduced infarct size (Figure 4H,J) in comparison with administration of RBCs from WT mice. RBCs from *ARG1* KO mice protected the isolated hearts from ischemia–reperfusion injury also after the RBCs were passed through a leukocyte filter (Supplemental Figure S10). The cardioprotective effect of RBCs from *ARG1* KO mice was abolished by the NOS inhibitor L-NAME (Figure 4D,E) and the sGC inhibitor ODQ (Supplemental Figure S11). Collectively, these results suggest that arginase 1 in RBC plays a critical role in the development of cardiac ischemia–reperfusion injury by regulating NO signaling.

### 3.5. Targeting Arginase 1 Improves Cardiac Tolerance to Ischemia–Reperfusion in T2D

**Figure 5:**
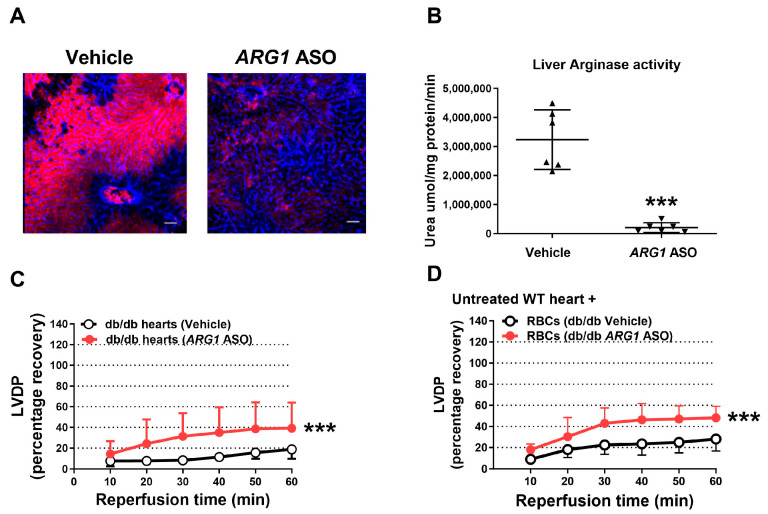
Targeting arginase 1 improves cardiac tolerance to ischemia–reperfusion in T2D. (**A**) Immunohistochemical picture of liver from *db*/*db* mice given vehicle or *ARG1* ASO for 6 weeks. Sections were stained with anti-arginase 1 (red) and anti-CD31 (blue). The bar indicates 50 mm. (**B**) Hepatic arginase activity in *db*/*db* mice treated with vehicle or *ARG1* ASO. (**C**,**D**) Recovery of LVDP in isolated mouse hearts subjected to 40 min ischemia and 60 min reperfusion. (**C**) Hearts from *db*/*db* mice treated with *ARG1* ASO (*n* = 5) or vehicle (*n* = 5). (**D**) RBCs from *db*/*db* mice treated with *ARG1* ASO (*n* = 5) or vehicle (*n* = 8) were given to isolated WT hearts at the onset of ischemia. Data are presented as means and SD. *** *p* < 0.001 denotes significant differences from the vehicle by an unpaired *t*-test (**B**) and by two-way ANOVA (**C**,**D**) Abbreviations as in Figure 1.

Previous studies have suggested that cardiac tolerance to ischemia–reperfusion in T2D is decreased, and that this is associated with increased arginase 1 expression and activity [12,35]. Therefore, specific inhibition of arginase 1 may be beneficial to improve cardiac tolerance to ischemia–reperfusion in T2D. This was tested using *ARG1* knockdown by ASO in *db*/*db* mice. Following administration of *ARG1* ASO to *db*/*db* mice for 6 weeks, arginase 1 expression and activity in the liver were significantly reduced (Figure 5A,B). *ARG1* ASO did not significantly affect body weight or blood glucose, although there was a trend towards decreased body weight after 5 weeks of treatment (Supplemental Figure S12). *ARG1* ASO did not alter cardiac contractility, heart rate (Supplemental Figure S13A,B), systolic or diastolic cardiac dimensions (Supplemental Figure S13C,D) or left ventricular wall thickness (Supplemental Figure S13E,F). Importantly, following *ARG1* knockdown, cardiac recovery of LVDP following ischemia–reperfusion in hearts from *db*/*db* mice was significantly improved and reached the same level as that observed in hearts from WT mice (Figure 5C). In addition, administration of RBCs from *db*/*db* mice treated with *ARG1* ASO significantly improved cardiac ischemic tolerance (Figure 5D).

## 4. Discussion

In the present study, we explored the distinct roles of arginase 1 and arginase 2 in cardiac ischemia–reperfusion injury. Our data show that arginase 1, but not arginase 2, plays a pivotal role in limiting tolerance to cardiac ischemia–reperfusion. Using genetically modified mouse models and pharmacological interventions, we demonstrate that arginase 1 in RBCs contributes to ischemia–reperfusion injury by attenuating NO signaling in both female and male mice. Importantly, suppression of arginase 1 restores cardiac tolerance to ischemia–reperfusion in T2D, demonstrating its potential as a therapeutic target for cardiovascular complications associated with T2D.

It is well established that both arginase isoforms are expressed in the cardiovascular system, including the vasculature and the myocardium, and that arginase expression is upregulated in cardiovascular disease [8,36,37,38,39]. Increased arginase expression in cardiac ischemia has been shown to contribute to ischemia–reperfusion injury and to aggravate ischemic injury in T2D [40,41,42]. Consequently, inhibition of arginase reduced infarct size in experimental models of ischemia–reperfusion via increased NO signaling [3,23]. Furthermore, a clinical study showed that inhibition of arginase protected from endothelial dysfunction induced by ischemia–reperfusion in patients with coronary artery disease [43]. Thus, available data clearly suggests important roles of arginase in cardiovascular disease and T2D, as well as potential beneficial therapeutic effects of targeting arginase in these diseases. However, due to the lack of isoform-specific pharmacological tools, it has remained unclear which isoform of arginase that is involved in these processes and which isoform to target therapeutically. By using ASO to knock down *ARG1* and *ARG2*, we show that the downregulation of arginase 1, but not arginase 2, increases cardiac tolerance to ischemia–reperfusion in the ex vivo heart model, clearly supporting an important role of arginase 1. The key role of arginase 1 was further established in vivo using conditional *ARG1* KO mice in the myocardial infarction model in which deletion of arginase 1 in hematopoietic and endothelial cells resulted in a reduction in infarct size. Interestingly, a similar cardioprotective effect was obtained using the arginase inhibitor nor-NOHA, suggesting that arginase 1 is the target for pharmacological arginase inhibition in this setting. Our data also reveals that the cardioprotective effect of *ARG1* deletion was dependent on NOS, demonstrated by the inhibitory effect of L-NMMA. This is in line with previous observations showing that the beneficial effect of pharmacological arginase inhibition is NOS-dependent [12,23] and supports the notion that arginase is a critical regulator of NO bioactivity [44,45,46].

Upregulation of arginase has been shown to be of particular relevance in T2D. Specifically, arginase has been suggested to contribute to increased cardiac injury during myocardial infarction and to endothelial dysfunction in T2D [12,21,44]. Targeting arginase has therefore been proposed as a potential beneficial therapy to attenuate cardiovascular complications in T2D. To improve the therapeutic precision by targeting arginase, we investigated the therapeutic effect of arginase 1 downregulation using ASO in *db*/*db* mice. Knockdown of *ARG1* restored cardiac tolerance to ischemia–reperfusion in hearts from *db*/*db* mice. Interestingly, RBCs from *ARG1* ASO *db*/*db* mice improved cardiac tolerance to ischemia–reperfusion in comparison with RBCs from untreated *db*/*db* mice. This finding suggests that the beneficial effect of pharmacological inhibition of arginase in RBC from *db*/*db* mice ex vivo led to cardiac protection against ischemia–reperfusion injury [12]. Collectively, our data suggests that targeting arginase 1 in RBCs is of benefit to attenuate cardiovascular injury in T2D. Development of such therapy would be of potentially great benefit considering that therapeutic strategies specifically targeting cardiovascular complications beyond glucose-lowering therapy are currently lacking.

As the *ARG1* KO mouse model lacks arginase 1 in both hematopoietic and endothelial cells, the cellular origin of arginase 1 as a regulator of ischemia–reperfusion injury was investigated using the isolated heart model and RBCs from these mice. We demonstrated that arginase 1 in RBCs but not in endothelial cells is a potential therapeutic target in myocardial ischemia–reperfusion. The recovery of cardiac function after ischemia was not affected in isolated buffer-perfused hearts from *ARG1* KO mice, which is consistent with our previous findings that pharmacological inhibition of cardiac arginase failed to improve tolerance to cardiac ischemia–reperfusion [23]. By contrast, administration of RBCs from *ARG1* KO mice markedly improved tolerance to cardiac ischemia–reperfusion, supporting a key regulatory role of arginase 1 in RBCs. This suggests that the cardioprotective effect observed in vivo is due to the deficiency of RBC-derived arginase 1 rather than endothelial cell arginase 1. The finding that isolated RBCs from *ARG1* KO improved cardiac tolerance to ischemia–reperfusion in isolated hearts indicates a potential therapeutic role of RBC arginase 1 inhibition in ischemic heart disease. Interestingly, downregulation of RBC arginase 1 induced cardioprotection in both sexes, suggesting sex-independent therapeutic efficacy of targeting RBC arginase 1.

It is in this context of interest that Heuser et al. [34] recently reported data from experiments using an RBC-specific *ARG1* KO mouse model. They did not find any difference in infarct size between the KO mice and WT controls following coronary artery ligation and reperfusion in vivo. These data, which suggest that RBC arginase 1 is not of importance in myocardial infarction, may appear to contrast our data. However, the WT littermate mice in the study by Heuser et al. [34] had very low or even undetectable levels of arginase in the RBC which makes the comparison with the KO mice difficult. By contrast, the WT mice in our study had clear and reproducible RBC arginase activity that was markedly lower in the KO model. The differences between the studies underscore the complexity of the system and make it challenging to definitively attribute the observed protective effects to RBCs alone. It remains possible that the benefits seen in arginase*-*deficient RBCs are secondary to arginase 1 depletion in other blood cells. Nonetheless, targeting RBC arginase 1 remains a promising avenue for future therapeutic investigation.

Unexpectedly, hearts from *ARG1* ASO-treated mice exhibited greater tolerance to ischemia–reperfusion than those from *ARG1* KO mice. This discrepancy may reflect differences between the life-long absence of arginase 1 in the KO model and the acute downregulation achieved with ASO treatment. Acute arginase inhibition has been shown to enhance cardiac protection in vivo through increased NO bioavailability [33]. Such effects may result from systemic arginase 1 downregulation in non-cardiac tissues, such as the liver and kidney, suggesting that the observed cardioprotection could stem from elevated NO bioavailability originating from remote organs rather than from direct cardiac effects [47]. By contrast, the life-long absence of arginase 1 in the KO model failed to confer cardiac protection in the isolated heart preparation, indicating that NO bioavailability might remain unchanged under these conditions. Interestingly, endothelial cell-specific *ARG1* deletion has been associated with eNOS downregulation, implying a potential functional crosstalk between endothelial arginase 1 and eNOS [48]. This interplay warrants further investigation in future studies.

Arginase 1 is a critical hepatic enzyme in the urea cycle that converts L-arginine to urea and ornithine for the disposal of ammonia. Lack of hepatic arginase 1 leads to hyperargininemia, a disorder with progressive mental impairment, growth retardation, spasticity and sometimes fatal episodes of hyperammonemia. This phenomenon is further confirmed by the observations that global *ARG1* KO mice have severe symptoms of hyperammonemia and die between postnatal days 10 and 14 [49]. Thus, systemic deficiency of arginase 1 should be avoided. Following the knockdown of *ARG1* using ASO treatment, we observed a marked downregulation of arginase 1 in the liver. However, we did not observe any obvious phenotype. This may be due to a large overcapacity of arginase 1 in the liver. However, longer observation times after *ARG1* knockdown are needed to exclude any side effects of the treatment. Nevertheless, the beneficial therapeutic effect of the downregulation of arginase 1 for the prevention of cardiac ischemic injury is promising and this issue deserves further investigation.

**Clinical implications and importance**

This study identifies RBC arginase 1 as a precise and clinically actionable therapeutic target for addressing the unmet need for cardioprotection in T2D. We demonstrate that selective inhibition of RBC arginase 1 restores NO-dependent cardiac protection to ischemia–reperfusion injury, directly correcting a central pathological mechanism in diabetic cardiovascular complications. The translational relevance is strengthened by the effectiveness of a clinically feasible ASO approach and by the robust protection observed in both sexes. Thus, targeting RBC arginase 1 offers a novel, mechanism-based strategy that extends beyond metabolic control to directly protect the diabetic heart by enhancing NO signaling.

**Limitations**

There are certain limitations in the study. First, although there was no clear phenotype observed and the general condition of the mice was unaffected, amino acid levels or ammonia accumulation were not determined in the *ARG1* ASO-treated mice. Furthermore, in vivo studies should be performed to investigate how levels of amino acids are affected long-term by the dose of *ARG1* ASO used in the study. Second, in vivo experiments of myocardial infarction were not performed with the ASO-treated mice due to a limited supply of the ASO. Experiments with isolated hearts and RBCs ex vivo were given priority to obtain detailed information regarding the effect of the arginase isoforms in the heart and RBCs. All RBCs from the ASO-treated animals were needed for the isolated heart studies so arginase activity and expression in RBCs from these animals could not be analyzed. Third, this study was conducted exclusively in mice. From a translational perspective, future investigations should include additional species and ideally multicenter collaborations to validate and extend these findings. Finally, potential off-target effects need to be considered. The ASO sequences were designed for high target specificity, and in vivo monitoring during our experiments revealed no signs of overt toxicity. Although these observations suggest a low risk of acute off-target effects under our experimental conditions, a systemic assessment would be an importance of future studies.

**Conclusions**

By using a combination of genetic and pharmacological approaches, the present study demonstrates that arginase 1, and not arginase 2, plays an important role in limiting cardiac tolerance to ischemia–reperfusion and increased cardiac vulnerability in T2D. The data supports a key role of RBC arginase 1, which via a NOS-sensitive mechanism modulates cardiac ischemic tolerance in T2D. Downregulation of RBC arginase 1 improves cardiac tolerance to ischemia–reperfusion and provides a promising novel therapeutic strategy for ischemic heart disease in T2D.

## Data Availability

The original contributions presented in this study are included in the article/ Supplementary Materials. Further inquiries can be directed to the corresponding author.

## Supplementary Materials

The following supporting information can be downloaded at: https://www.mdpi.com/article/10.3390/antiox15010058/s1. The legends of the Supplementary Materials: Figure S1. Sheme of ARG1/2 ASO injection. Figure S2. Kidney sections of mice treated with *ARG1* antisense oligonucleotide (*ARG1* ASO) or vehicle for 6 weeks. Sections of the kidney were stained with anti-arginase 2. Bar indicates 50 mm. Figure S3. Body weight of mice given vehicle (*n* = 6) or arginase 1 anti-sense oligonucleotide (*ARG1* ASO, *n* = 6) for 6 weeks. Figure S4. Mouse cardiac function by transthoracic echocardiography after 6-week treatment with vehicle or *ARG1* anti-sense oligonucleotide (*ARG1* ASO). (A) Heart rate; (B) Percentage of fractional shortening; (C) Systolic diameter; (D) Diastolic dimeter; (E) Interventricular septum; (F) Posterior wall thickness. Figure S5. Western blot of (A) arginase 2 and (B) arginase 1 in mouse kidney tissue following 6 weeks of *ARG2* ASO or vehicle treatment. GAPDH was used as loading control. Figure S6. Body weight of mice given vehicle (*n* = 10) or arginase 2 antisense oligonucleotide (*ARG2* ASO, *n* = 10) for 6 weeks. Figure S7. Arginase 1 anti-sense oligonucleotide reduces arginase 1 expression and induces cardiac protection. After 6 weeks treatment of vehicle or arginase 1 (*ARG1*) or arginase 2 (*ARG2*) anti-sense oligonucleotide (ASO), recovery of left ventricular developed pressure (LVDP) in isolated hearts subjected to 40 min ischemia and 60 min reperfusion. (A) Buffer-perfused hearts from *ARG1* ASO-treated mice (*n* = 5) or vehicle (*n* = 10) and *ARG2* ASO-treated mice (*n* = 6) or vehicle (*n* = 7). (D) RBCs from *ARG1* ASO-treated mice (*n* = 9) or vehicle (*n* = 5) and *ARG2* ASO-treated mice (*n* = 6) or vehicle (*n* = 7) were administered at ischemia onset to isolated hearts. Data are shown as mean ± SD. * *p* < 0.05 and *** *p* < 0.001 denote significant differences vs. vehicle, analyzed by unpaired t-test (B) or two-way ANOVA (C, D). Figure S8. *ARG1* mRNA in bone marrow cells of *ARG1* wild type (*ARG1* WT) and conditional knockout (*ARG1* KO) mice. Figure S9. Arginase activity was detected in isolated RBCs from *ARG1* wild type (*ARG1* WT) and ARG1 conditional knockout (*ARG1* KO) mice. ** *p* < 0.01 denotes significant differences from vehicle using an unpaired t-test. Figure S10. Recovery of left ventricular developed pressure (LVDP) in isolated mouse hearts subjected to 40 min ischemia and 60 min reperfusion. RBCs from *ARG1* WT (*n* = 10) or *ARG1* KO (*n* = 8) mice after white blood cell filter were given to isolated and perfused WT hearts at the onset of ischemia. *** *p* < 0.001 denotes significant differences to the vehicle group by two-way ANOVA. Figure S11. Recovery of left ventricular developed pressure (LVDP) in isolated mouse hearts subjected to 40 min ischemia and 60 min reperfusion. RBCs from male *ARG1* WT (*n* = 7) or *ARG1* KO (*n* = 8) mice, with or without sGC inhibitor (ODQ, 5uM, *n* = 4), were given to isolated and perfused WT hearts at the onset of ischemia. *** *p* < 0.001 denotes significant differences to the vehicle group by two-way ANOVA. Figure S12. (A) Body weight and (B) Glucose level (*n* = 5) of db/db mice given vehicle (*n* = 10) or arginase 1 anti-sense oligonucleotide (*ARG1* ASO, *n* = 10) for 6 weeks. Figure S13. Mouse (db/db) cardiac function by transthoracic echocardiography after 6-week treatment of vehicle or *ARG1* anti-sense oligonucleotide (*ARG1* ASO). (A) Heart rate; (B) Percentage of fractional shortening; (C) Diastolic diameter; (D) Systolic diameter; (E) Interventricular septum; (F) Posterior wall thickness. Supplemental Table S1. Heart rate in mice subjected to in vivo myocardial IR. MI: myocardial ischemia; MR: myocardial reperfusion; mice with preserved arginase 1 obtained saline (Arg1 WT, *n* = 6) or arginase inhibitor nor-NOHA (*ARG1* WT+nor-NOHA), *n* = 5); mice with arginase 1 deleted in RBCs and endothelium were given either saline (*ARG1* KO, *n* = 6) or NOS inhibitor L-NMMA (*ARG1* KO+L-NMMA, *n* = 5). Data are presented as mean ± SD.

## Abbreviations

The following abbreviations are used in this manuscript:

ASO	antisense oligonucleotides
ARG	arginase
EC	endothelial cells
KH	Krebs–Henseleit
KO	knockout
L-NAME	N-Nitro-L-arginine methylester
L-NMMA	N^G^-monomethyl-L-arginine monoacetate
LVDP	left ventricular developed pressure
NO	Nitric Oxide
nor-NOHA	N^ꞷ^-hydroxy-nor-L-arginine
NOS	Nitric oxide synthase
ODQ	1H- [1,2,4] oxadiazolo [4,3,-a] quinoxalin-1-one
RBC	red blood cell
sGC	soluble guanylate cyclase
T2D	type 2 diabetes
WT	wild type
