# Supplementary material for: Targeting Arginase 1 but Not Arginase 2 Protects from Myocardial Ischemia–Reperfusion Injury via Nitric Oxide Signaling by Red Blood Cells in Type 2 Diabetes
Source: Antioxidants (Basel). 2026 Jan 1;15(1):58. doi: 10.3390/antiox15010058 (PMC12837884; doi:10.3390/antiox15010058)
Supplement: Supplementary file 1 [file antioxidants-15-00058-s001.zip › antioxidants-4030200-supplementary.pdf]

Supplementary Figures

Suppl. Figure S1

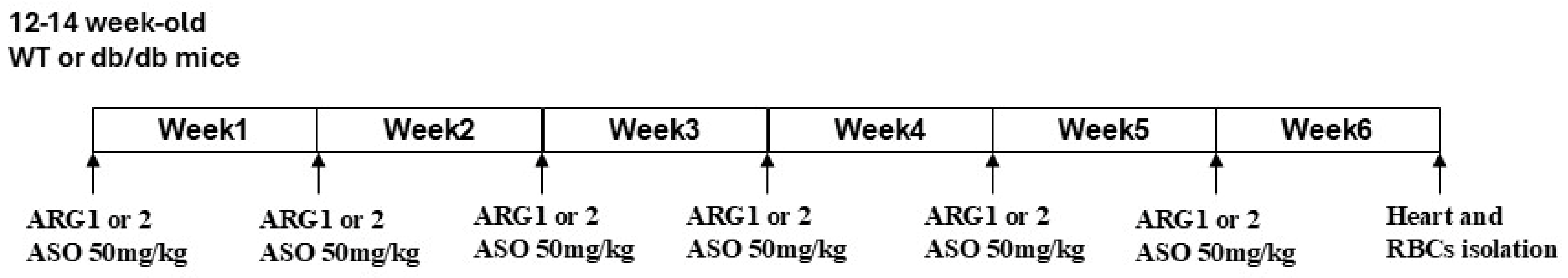

Figure S1. Scheme of ARG1/2 ASO injection

Suppl.
Figure S2

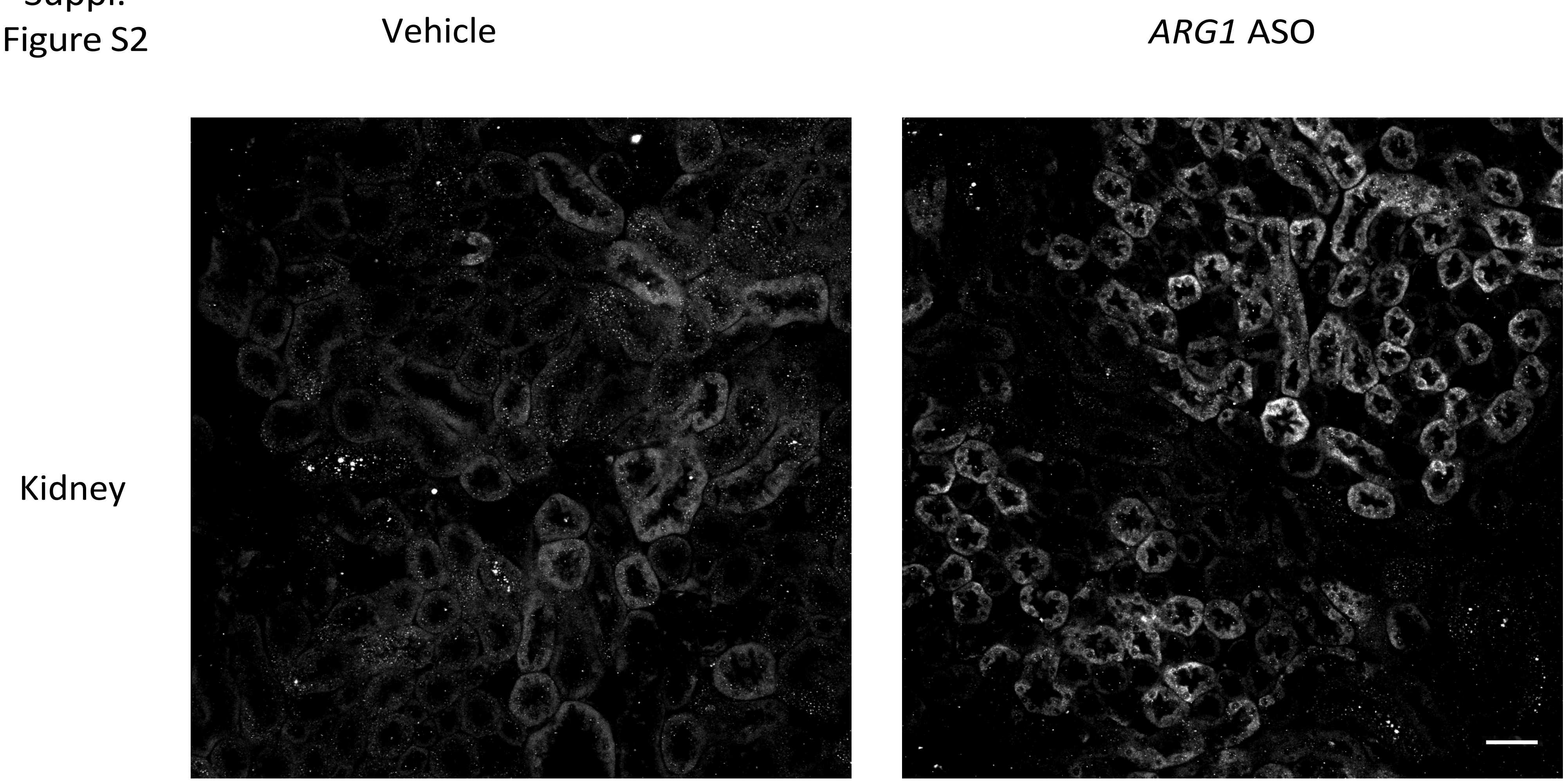

Figure S2. Kidney sections of mice treated with *ARG1* antisense oligonucleotide (*ARG1* ASO) or vehicle for 6 weeks. Sections of the kidney were stained with anti-arginase 2. Bar indicates 50 mm.

Suppl. Figure S3

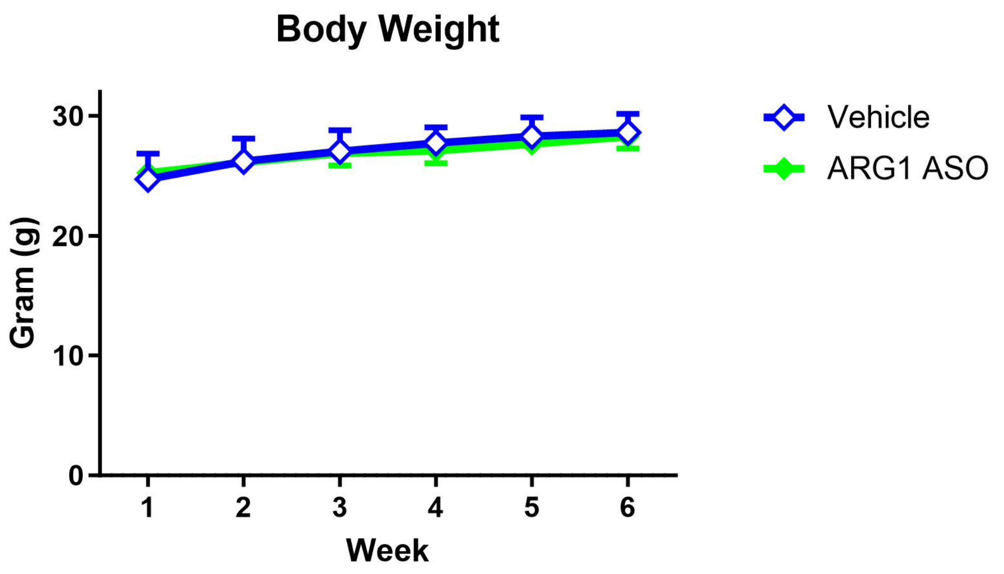

Figure S3. Body weight of mice given vehicle (n=6) or arginase 1 anti-sense oligonucleotide (*ARG1* ASO, n=6) for 6 weeks.

Suppl. Figure S4.

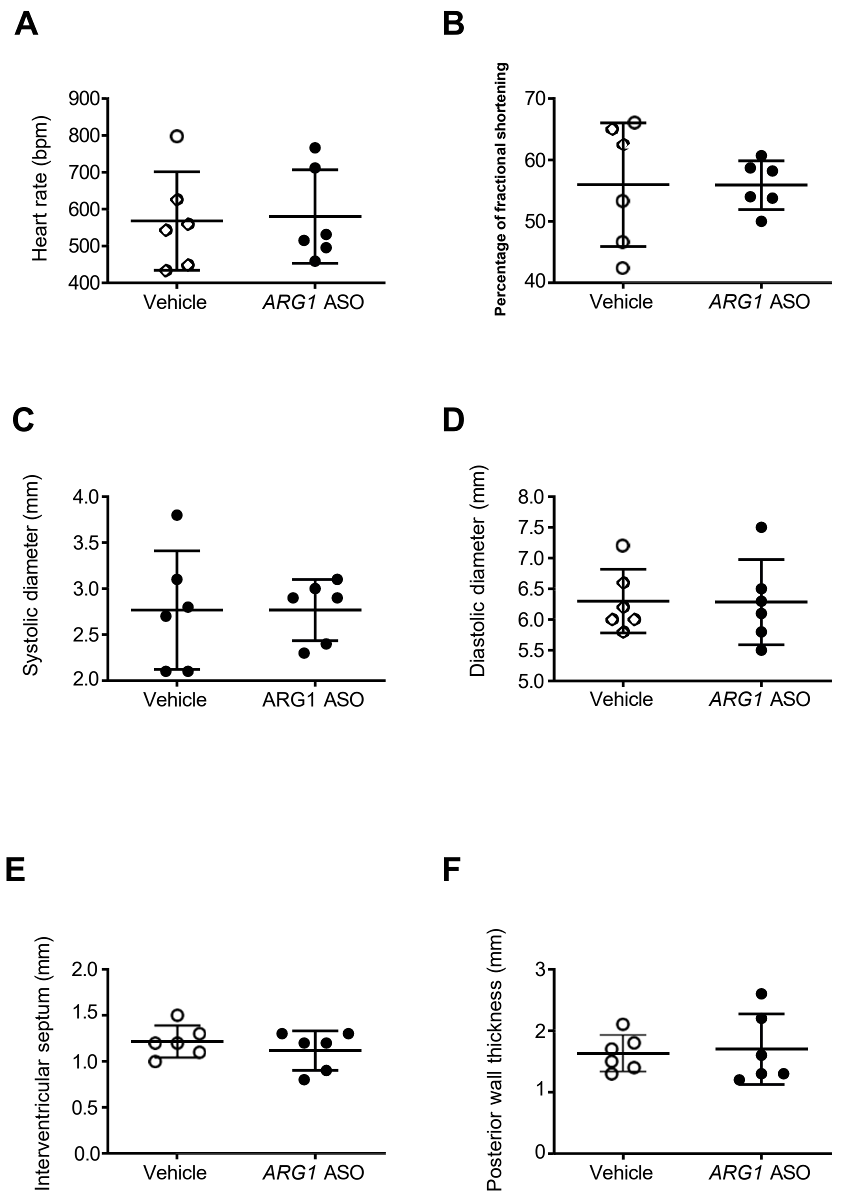

Figure S4. Mouse cardiac function by transthoracic echocardiography after 6-week treatment with vehicle or *ARG1* anti-sense oligonucleotide (*ARG1* ASO). (A) Heart rate; (B) Percentage of fractional shortening; (C) Systolic diameter; (D) Diastolic dimeter; (E) Interventricular septum; (F) Posterior wall thickness.

Suppl. Figure S5

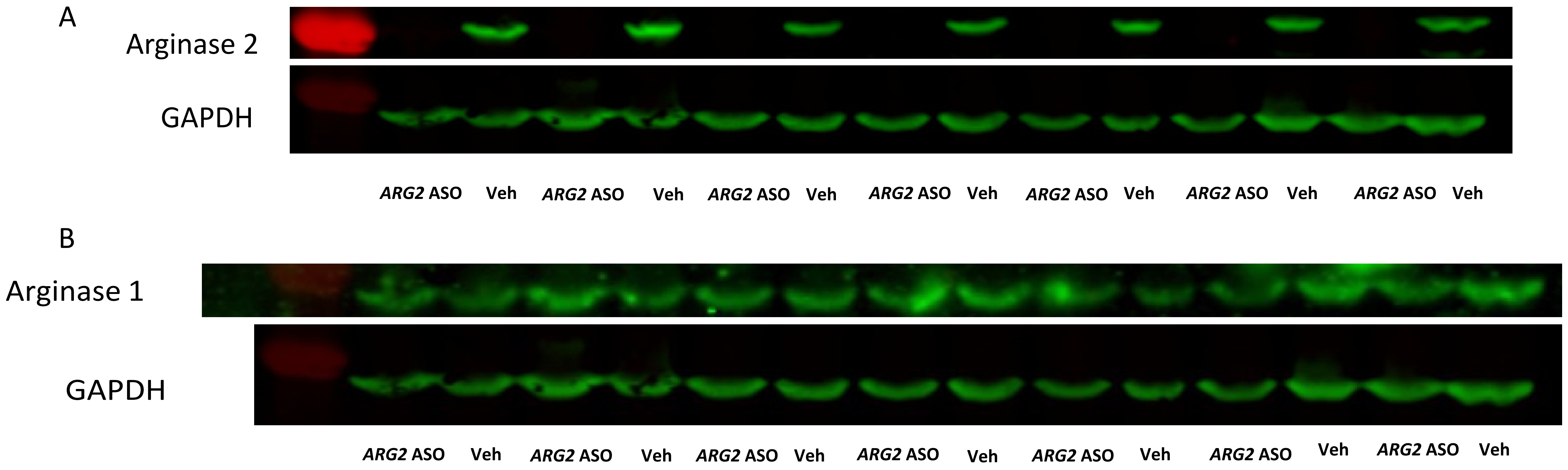

Figure S5. Western blot of (A) arginase 2 and (B) arginase 1 in mouse kidney tissue following 6 weeks of *ARG2* ASO or vehicle treatment. GAPDH was used as loading control.

Suppl. Figure S6

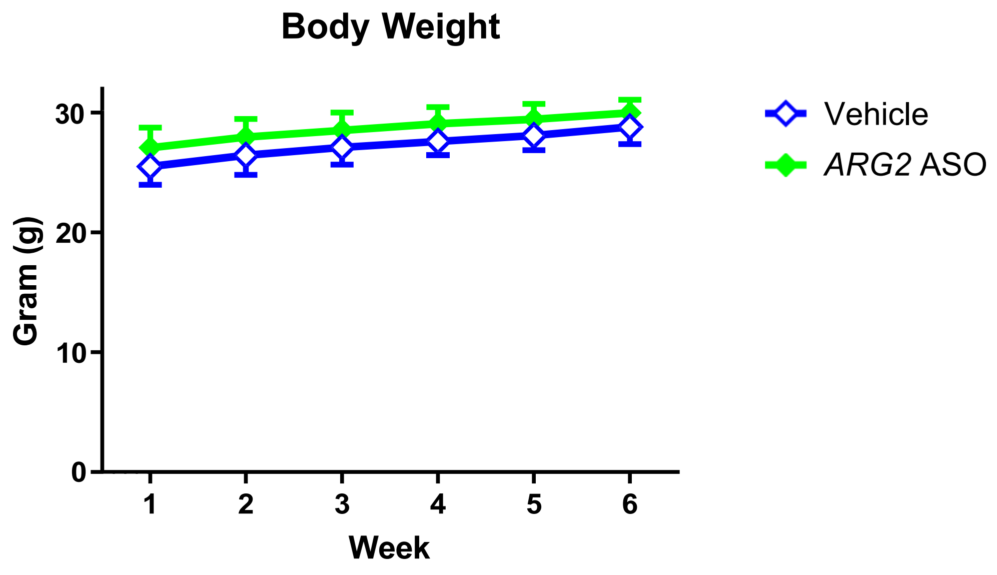

Figure S6. Body weight of mice given vehicle (n=10) or arginase 2 antisense oligonucleotide (*ARG2* ASO, n=10) for 6 weeks.

Suppl. Figure S7

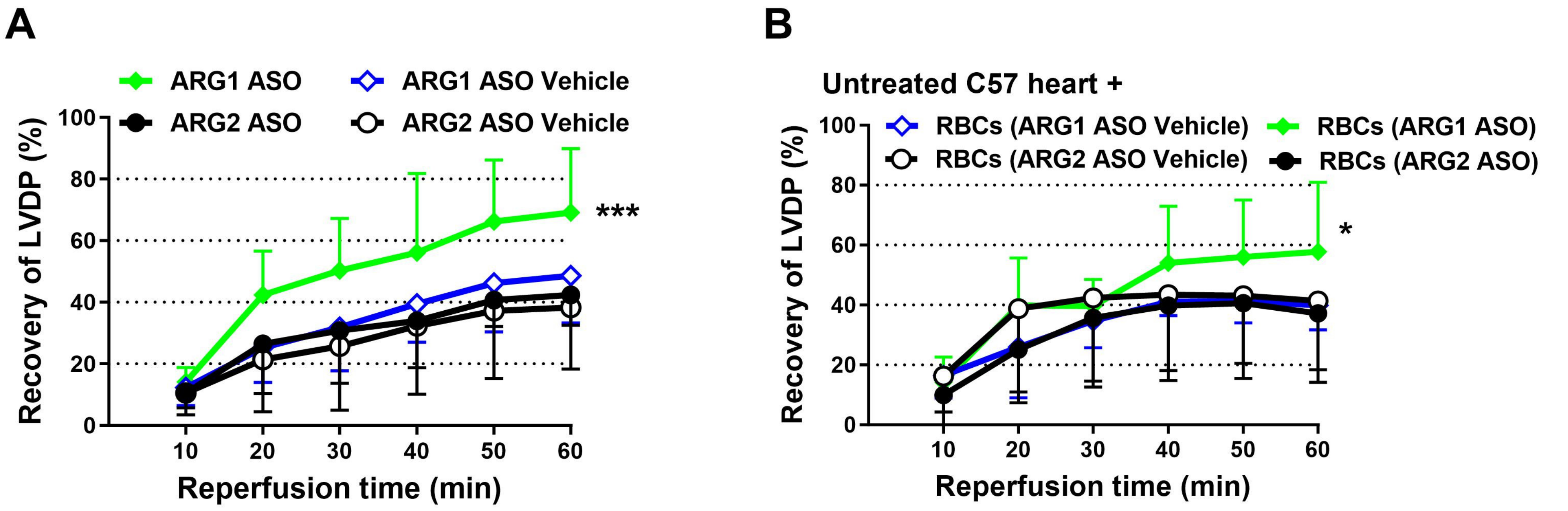

Figure S7. **Arginase 1 anti-sense oligonucleotide reduces arginase 1 expression and induces cardiac protection.** After 6 weeks treatment of vehicle or arginase 1 (*ARG1*) or arginase 2 (*ARG2*) anti-sense oligonucleotide (ASO), recovery of left ventricular developed pressure (LVDP) in isolated hearts subjected to 40 min ischemia and 60 min reperfusion. (A) Buffer-perfused hearts from *ARG1* ASO-treated mice (n=5) or vehicle (n=10) and *ARG2* ASO-treated mice (n=6) or vehicle (n=7). (D) RBCs from *ARG1* ASO-treated mice (n=9) or vehicle (n=5) and *ARG2* ASO-treated mice (n=6) or vehicle (n=7) were administered at ischemia onset to isolated hearts. Data are shown as mean ± SD. **P*<0.05 and ****P*<0.001 denote significant differences vs. vehicle, analyzed by unpaired t-test (B) or two-way ANOVA (C, D).

Suppl. Figure S8

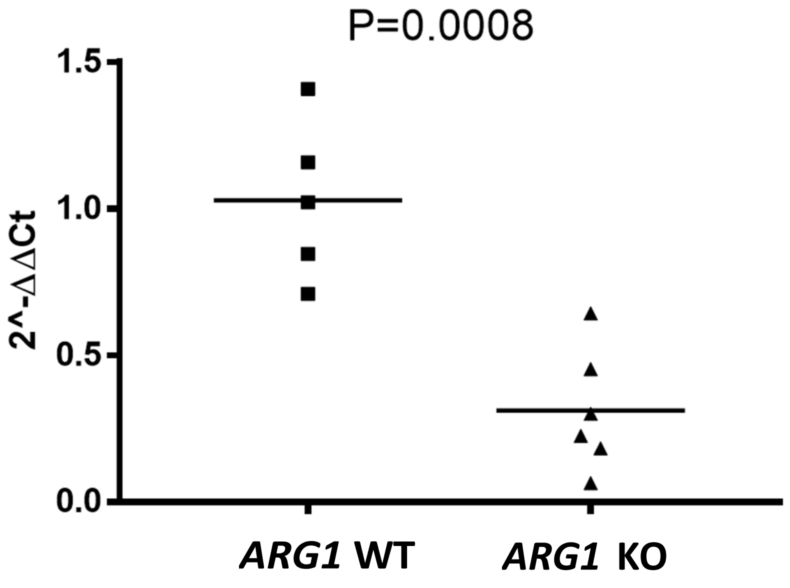

Figure S8. *ARG1* mRNA in bone marrow cells of *ARG1* wild type (*ARG1* WT) and conditional knockout (*ARG1* KO) mice.

Suppl. Figure S9

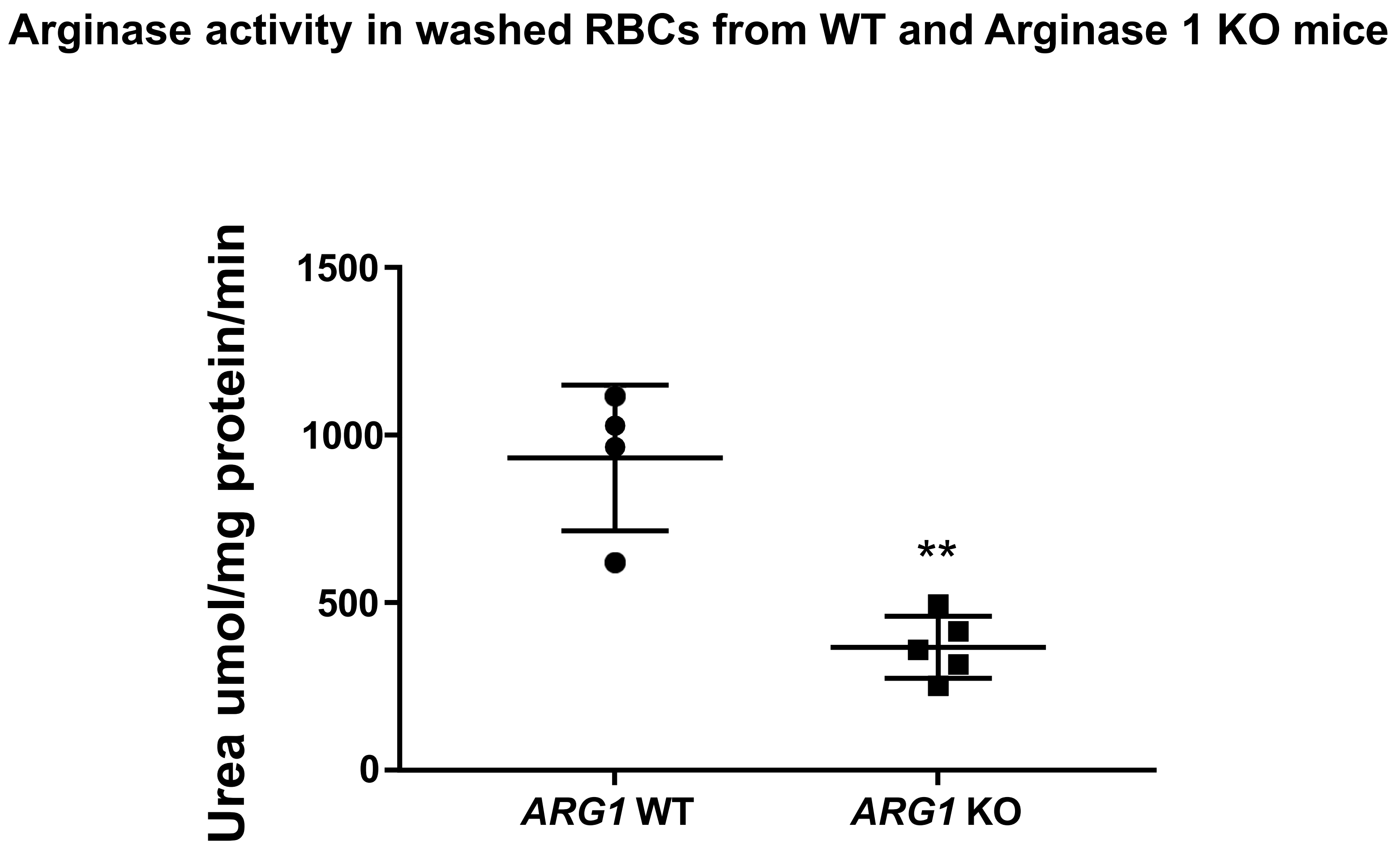

Figure S9 Arginase activity was detected in isolated RBCs from *ARG1* wild type (*ARG1* WT) and ARG1 conditional knockout (*ARG1* KO) mice. ***P*<0.01 denotes significant differences from vehicle using an unpaired t-test.

Suppl.
Figure S10

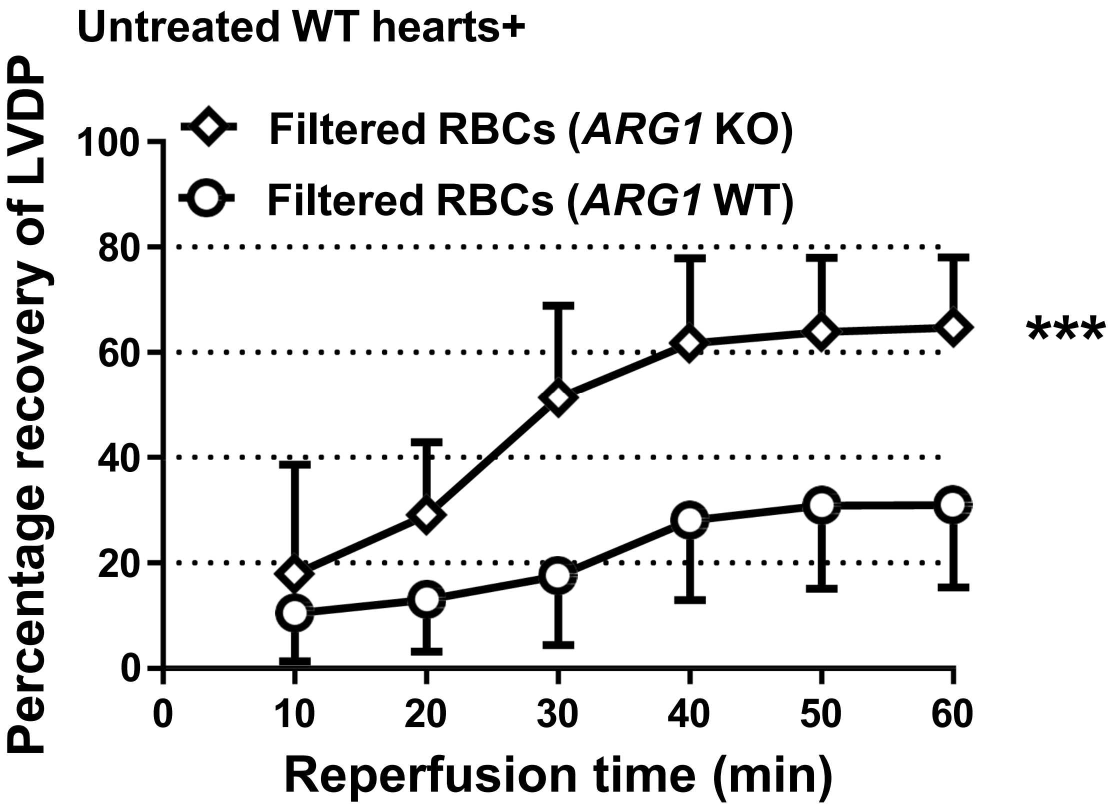

Figure S10. Recovery of left ventricular developed pressure (LVDP) in isolated mouse hearts subjected to 40 min ischemia and 60 min reperfusion. RBCs from *ARG1* WT (n=10) or *ARG1* KO (n=8) mice after white blood cell filter were given to isolated and perfused WT hearts at the onset of ischemia. ****P*<0.001 denotes significant differences to the vehicle group by two-way ANOVA

Suppl.
Figure S11

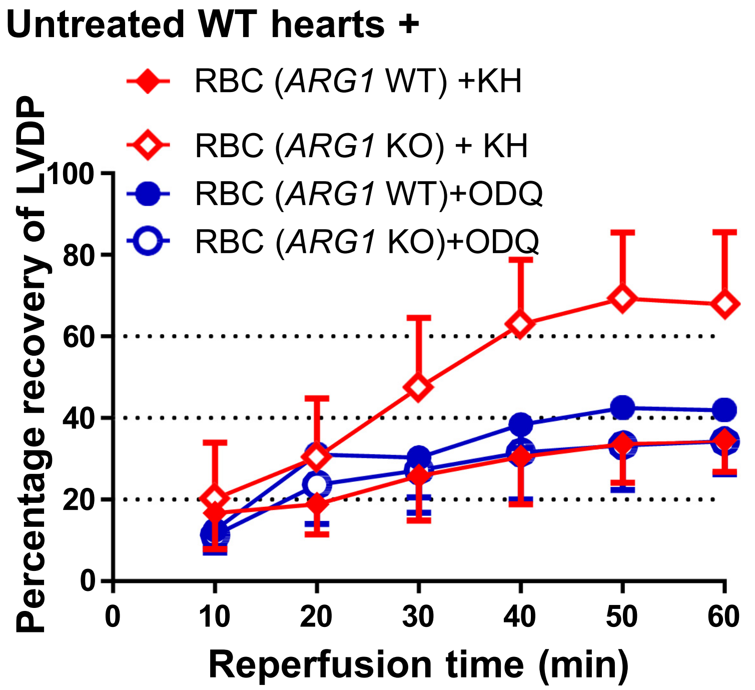

Figure S11. Recovery of left ventricular developed pressure (LVDP) in isolated mouse hearts subjected to 40 min ischemia and 60 min reperfusion. RBCs from male *ARG1* WT (n=7) or *ARG1* KO (n=8) mice, with or without sGC inhibitor (ODQ, 5uM, n=4), were given to isolated and perfused WT hearts at the onset of ischemia. ****P*<0.001 denotes significant differences to the vehicle group by two-way ANOVA

Suppl. Figure S12

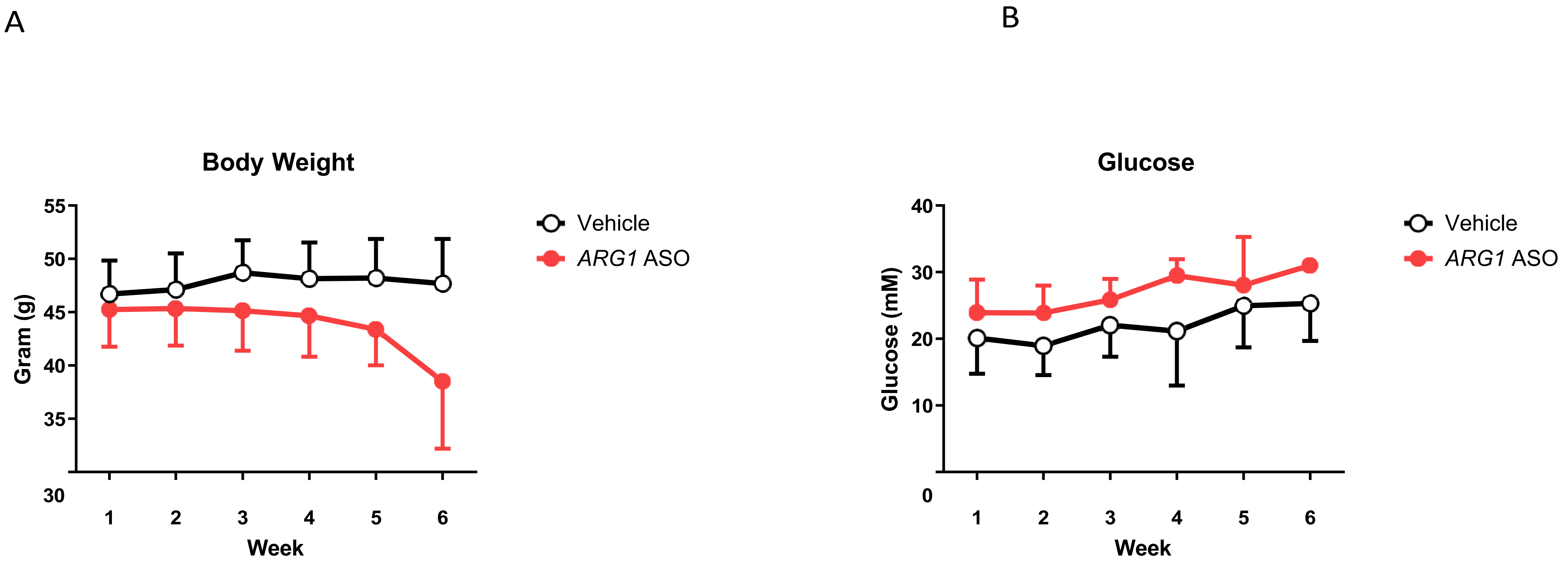

Figure S12. (A)Body weight and(B) Glucose level (n=5) of db/db mice given vehicle (n=10) or arginase 1 anti-sense oligonucleotide (*ARG1* ASO, n=10) for 6 weeks.

Suppl. Figure S13

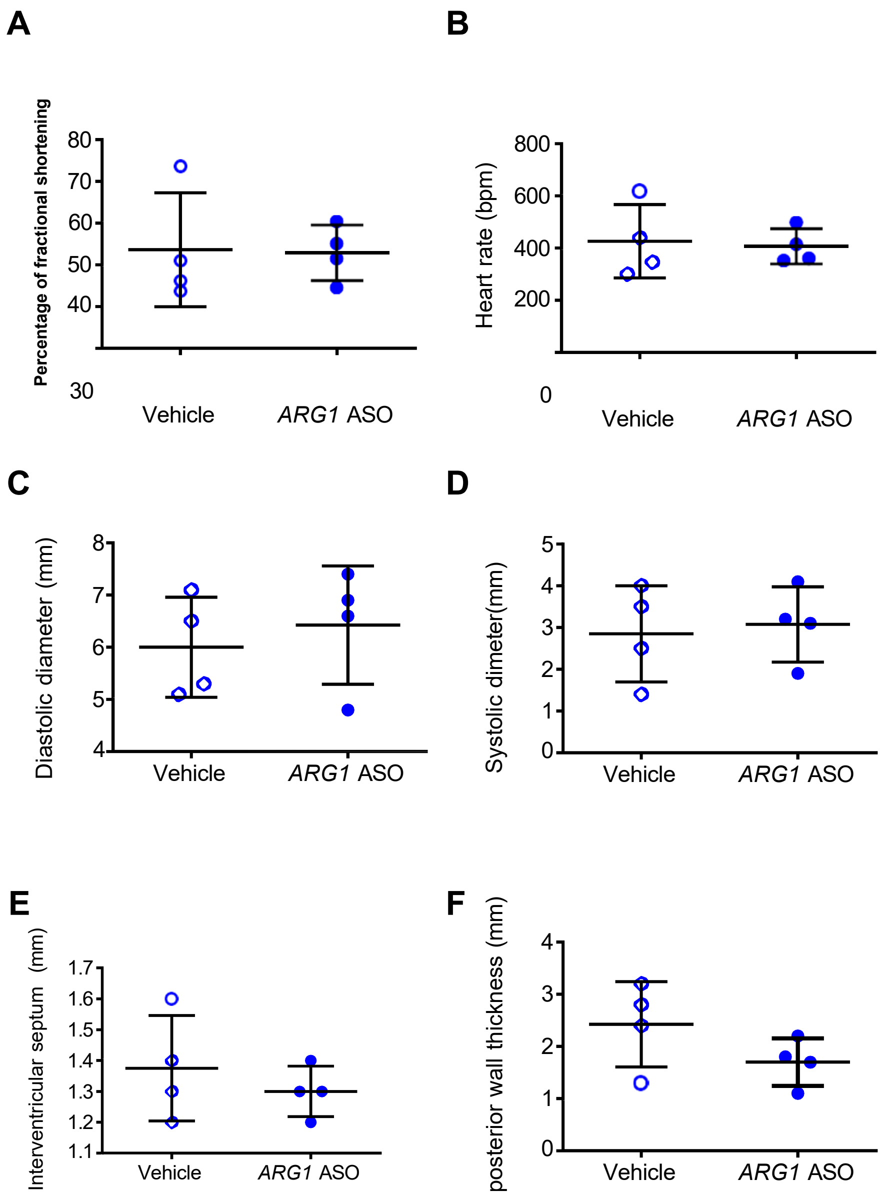

Figure S13. Mouse (db/db) cardiac function by transthoracic echocardiography after 6-week treatment of vehicle or *ARG1* anti-sense oligonucleotide (*ARG1* ASO). (A) Heart

rate; (B) Percentage of fractional shortening; (C) Diastolic diameter; (D) Systolic diameter; (E) Interventricular septum; (F) Posterior wall thickness.

Suppl. Table S1. Heart rate in mice subjected to in vivo myocardial IR.

Group	15 min before MI	Before MI	Before MR	15 min MR	30 min MR	60 min MR	90 min MR	120 min MR
ARG1 WT	432±47	423±36	429±37	431±36	437±43	427±16	446±38	415±17
ARG1 KO	406±25	422±24	438±42	447±31	457±32	420±22	445±37	429±30
ARG1 WT nor-NOHA	412±37	425±37	430±39	426±35	411±34	421±19	426±20	403±32
ARG1 KO L-NMMA	415±19	449±23	451±39	447±31	456±36	439±36	421±32	445±46

MI: myocardial ischemia; MR: myocardial reperfusion; mice with preserved arginase 1 obtained saline (Arg1 WT, n=6) or arginase inhibitor nor-NOHA (*ARG1* WT+nor-NOHA), n=5); mice with arginase 1 deleted in RBCs and endothelium were given either saline (*ARG1* KO, n=6) or NOS inhibitor L-NMMA (*ARG1* KO+L-NMMA, n=5). Data are presented as mean ± SD.
